# Downregulation of salusins alleviates hypertrophic cardiomyopathy via attenuating oxidative stress and autophagy

**DOI:** 10.1186/s40001-024-01676-z

**Published:** 2024-02-09

**Authors:** Jing-Yi Dang, Wei Zhang, Yi Chu, Jiang-Hong Chen, Zhao-Le Ji, Pin Feng

**Affiliations:** Department of Cardiology, Tangdu Hospital, Airforce Medical University, No. 569 Xinsid Road, Xi’an, 710038 China

**Keywords:** Salusin, Hypertrophic cardiomyopathy, Fibrosis, Oxidative stress, Autophagy

## Abstract

**Introduction:**

Salusins, which are translated from the alternatively spliced mRNA of torsin family 2 member A (TOR2A), play a vital role in regulation of various cardiovascular diseases. However, it remains unclear precisely regarding their roles in hypertrophic cardiomyopathy (HCM). Therefore, this study was conducted to explore therapeutic effect and the underlying mechanisms of salusins on HCM.

**Material and methods:**

In vivo experiments, Sprague–Dawley rats were used to induce HCM model by angiotensin (Ang) II infusion for 4 weeks. The rats were randomly divided into four groups, namely, Saline + Control shRNA (n = 7), Ang II + Control shRNA (n = 8), Saline + TOR2A shRNA (n = 7), and Ang II + TOR2A shRNA groups (n = 8). After HCM induction, doppler echocardiography is recommended to evaluate heart function. In vitro experiments, primary neonatal rat cardiomyocytes (NRCMs) and cardiac fibroblasts (NRCFs) were obtained from newborn rats, and were treated with Ang II (10^–6^ M) for 24 h.

**Results:**

After treatment with Ang II, levels of salusin-α and salusin-β were elevated in serum and cardiac tissues of rats and in the neonatal rat cardiomyocytes and cardiac fibroblasts. Downregulation of salusins alleviated the Ang II-induced cardiac hypertrophy by suppressing the increased atrial natriuretic peptide (ANP), brain natriuretic peptide (BNP) and beta-myosin heavy chain (β-MHC) and cardiac fibrosis by blocking collagen I, collagen III and transforming growth factor-beta (TGF-β), and it also attenuated oxidative stress by suppressing the increased reactive oxygen species (ROS) and malondialdehyde (MDA) levels and reversing the decreased superoxide dismutase (SOD) activity and autophagy by inhibiting the increased microtubule-associated protein light chain 3B (LC3B), Beclin1, autophagy related gene (Atg) 3 and Atg5 in the cardiac tissues of Ang II-infused rats and in the Ang II-treated NRCMs.

**Conclusions:**

All these findings suggest that the levels of salusins were elevated in the HCM, and targeting of salusins contributes to alleviation of cardiac hypertrophy and fibrosis probably via attenuating oxidative stress and autophagy. Accordingly, targeting of salusins may be a strategy for HCM therapy.

**Supplementary Information:**

The online version contains supplementary material available at 10.1186/s40001-024-01676-z.

## Introduction

Evidence from epidemiological studies has revealed an increased incidence and mortality of cardiovascular diseases (CVDs) year by year [1], which are the leading cause of death worldwide and pose serious impacts on social economy and human health [2–4]. Hypertrophic cardiomyopathy (HCM), which is characterized by asymmetric ventricular hypertrophy, is the most common inherited heart disease that occurs in various CVDs [5, 6]. HCM, which is the most common cardiac cause of sudden death in young population, can lead to functional disability from heart failure and stroke; However, most genetically and clinically affected individuals may remain undiagnosed, and lots of them do not suffer significantly decreased life expectancy or substantial symptoms [7].

Measurement of biomarkers has revolutionized the work-up of patients with suspected CVDs, in which the natriuretic peptides are the most widely used contemporary cardiovascular biomarkers in the diagnosis and prognosis of heart failure [8]. Salusins including salusin-α and salusin-β are translated from the alternative splicing at the C-terminal end of torsin family 2member A (TOR2A) [9]. Salusin-α, an endogenous biologically active polypeptide of 28 amino acids, and salusin-β, a novel bioactive peptide of 20 amino acids, are related to CVDs [10, 11]. More and more researches have demonstrated that salusin-α and salusin-β participate in the regulation of heart failure [12], cardiovascular remodeling [13], hypertension [14] and pulmonary arterial hypertension [15]. Oxidative stress is characterized as an imbalance between the production of reactive oxygen species (ROS) and elimination of oxidative stress [16]. Recently, the free radicals and ROS-induced oxidative stress [17] has been linked to the damage of DNA, proteins and lipids, consequently resulting in cell death [18]. More recently, accumulating evidence has revealed that an increase in ROS production is strongly related to various CVDs, including myocardial infarction [17], ischemia/reperfusion, heart failure and HCM [19, 20]. Notably, salusins are involved in oxidative stress of multiple diseases, including diabetic cardiomyopathy [21], diabetic nephropathy [22] and acute kidney injury [23]. Nevertheless, it remains unclear precisely whether salusins participate in oxidative stress of HCM.

Autophagy, an intracellular lysosomal degradative pathway, can maintain cellular homeostasis by rewiring cellular metabolism that connects dynamic catabolic to anabolic processes [24, 25]. Recently, lots of studies have revealed that abnormal autophagy in cardiac myocytes induces cardiomyocytes death and actively mediates cardiac damage and dysfunction under some situations, such as doxorubicin cardiomyopathy, reperfusion injury and myocardial infarction [26–28]. A recent study has confirmed that the accumulation of undigested autophagosomes results in cardiac dysfunction in lysosomal storage diseases [29]. Herein, we attempted to explore the therapeutic effect and the underlying mechanisms of salusins on HCM, and demonstrated that downregulation of salusins contributed to alleviating HCM possibly via attenuating oxidative stress and autophagy.

## Materials and methods

### Animals and rat model of hypertrophic cardiomyopathy

Forty-six Sprague–Dawley (SD) rats weighting 160–180 *g* (Male, 10–12 weeks old) were obtained from the Charles River (Beijing, China) and housed in a temperature-controlled room under a 12–12 h light–dark cycle, with standard chow and tap water ad libitum*.* All experiments and procedures involving animals in this study were approved by the appropriate Animal Care and Use Committee of Airforce Medical University (IACUC-21070267) and performed in strict accordance with the Guidelines for the Care and Use of Laboratory Animals (NIH publication No. 85–23, revised 1996).

### Experimental arrangement and drug

Firstly, SD rats were randomly divided into two groups, namely, saline and Ang II groups (n = 8 in each group). Secondly, SD rats were randomly divided into four groups, namely, Saline + Control shRNA (n = 7), Ang II + Control shRNA (n = 8), Saline + TOR2A shRNA (n = 7), and Ang II + TOR2A shRNA groups (n = 8). The rats in the Saline + TOR2A shRNA group and Ang II + TOR2A shRNA group were transduced adeno-associated viruses (AAVs, TOR2A shRNA, 2 × 10^11^ vg; OBIO, Shanghai, China) to downregulate salusins levels via tail-vein injection. Two weeks later, the rats were infused with Ang II (1.44 mg/kg/day [30]; Sigma, MO, USA) or saline (control group) with osmotic pumps for four consecutive weeks.

### Echocardiography for evaluating heart function

After 4 weeks of Ang II infusion, all rats were weighted and anesthetized with isoflurane (2.5–3.0%) to perform echocardiography using a 21-MHz probe ultrasound system (Visual Sonics, Toronto, Canada). Left ventricle (LV) ejection fraction (EF), fractional shortening (FS), LV weight (LW), LV anterior wall thickness at end systole (LVAWs), LV anterior wall thickness at end diastole (LVAWd), LV posterior wall thickness at end systole (LVPWs), LV posterior wall thickness at end diastole (LVPWd), interventricular septal thickness at end-systole (IVSs) and at end-diastole (IVSd) were calculated as the mean of three consecutive cardiac cycles using a computer algorithm. Thereafter, all rats were euthanized to collect the blood from the heart after sacrifice for the following detection of salusin levels and then the heart was rapidly excised for determination of heart weight (HW) and salusin levels as well as other procedures described below. Based on their body weight (BW) or tibia length (TL), the HW/BW, HW/TL and LW/BW were calculated.

### Enzyme-linked immuno sorbent assay (ELISA) for determining salusin levels

Whole blood obtained was placed at room temperature for 3–4 h and then serum was collected by centrifugation at 2000 rpm for 20 min. In addition, the cardiac tissues from rats in the four groups were collected and washed to remove the remaining blood with ice-cold phosphate buffered saline (PBS) (0.01 M, pH = 7.4). 200 mg cardiac tissue was homogenized in 2 ml ice-cold 1 × PBS, and centrifuged at 5000 × *g* for 5 min to get supernatant. The protein concentration was detected by BCA kit (Beyotime Biotecnology, Shanghai, China). Thereafter, the optical density values of serum and cardiac tissue's supernatant at 450 nm were separately detected on a microplate reader (BioTek, VT, USA) according to the ELISA Kit (Uscn Life Science Inc. Wuhan China) instructions and the test results were presented as pg/ml, with a minimum detectable concentration of 0.93 pg/ml for salusin-α and 1.75 pg/ml for salusin-β.

### Wheat germ (WGA) staining for evaluating myocardial cell hypertrophy

According to the manufacture’s instruction, left ventricular portion of heart was fixed in 10% neutral buffered formalin and then cut into 3–5 μm thick sections. Thereafter, the myocardial section was stained with 5 μg/ml WGA staining (Service Biological Technology Co., Ltd, Wuhan, China) and then with 4’,6-diamidino-2-phenylindole (DAPI; Life Technologies Co., NY, USA) for 3 min at room temperature in dark place to assess the size of cardiomyocytes and the degree of hypertrophic cardiomyocytes. Samples were imaged under a fluorescence microscope (Carl Zeiss GmbH, Oberkochen, Germany) and analyzed using Image J software (National Institutes of Health).

### Sirius red staining

Cardiac fibrosis was determined by sirius red staining (Service Biological Technology Co., Ltd, Wuhan, China). Briefly, the myocardial tissues of rats were fixed in 4% paraformaldehyde for 4 h, imbedded in paraffin and cut into 3–4 μm. After dewaxing with xylene and rehydration through graded ethanol, sirius red staining was carried out for collagen detection and then the entire section area was canned under an optical microscope (Carl Zeiss GmbH), and Image-Pro Plus software (Media Cybernetics Inc., MD, USA) was used to analyze the percent of total positive.

### Evaluation of oxidative stress

Rat myocardial tissues were thoroughly mixed with beads and PBS on a vortex mixer for 40 min, crushed to prepare 10% tissue homogenates (mg/μl), and then centrifuged at 5000 × *g* for 5 min to get the supernatant. Thereafter, the levels of ROS, malondialdehyde (MDA) and superoxide dismutase (SOD) were separately detected with dihydroethidium staining (Beyotime), lipid peroxidation assay kit (Beyotime, Shanghai, China), and SOD assay kit (Beyotime, Shanghai, China) in accordance with the manufacturer's instructions.

### Immunofluorescence

Paraformaldehyde (4%) was employed to fix the cardiac tissues for 24 h at room temperature. The samples were incubated with primary antibodies against microtubule-associated protein light chain 3B (LC3B; Abcam, MA, USA) or 8-hydroxy-2ʹ-deoxyguanosine (8-OHdG; Santa, TX, USA) for 12–15 h at 4 ℃, subsequently with relevant secondary antibodies (Abcam, MA, USA) at room temperature for 2 h. DAPI (Life Technologies Co., NY, USA) was used to counterstain the nucleus. Finally, the morphology of the nucleus was viewed under a fluorescence microscope (Carl Zeiss GmbH, Oberkochen, Germany) and analyzed using Image J software (National Institutes of Health). The strength of the positive expression was assessed according to the fluorescence intensity.

### Primary cell culture

Primary neonatal rat cardiomyocytes (NRCMs) and cardiac fibroblasts (NRCFs) were obtained from 1 to 3-day-old newborn SD rats (Airforce Medical University, Xi’an, China). Briefly, 30 newborn rats were euthanasia by cervical dislocation after anesthetized with 2% isoflurane for 2 min. The isolated left ventricle of heart was digested in PBS containing collagenase type II (Worthington, NJ, USA) and pancreatin (Sigma, MO, USA), then the cells were collected and cultured in Dulbecco modified Eagle medium (DMEM) (Gibco, Invitrogen Inc.) supplemented with 2 mM glutamine, 10% heat-inactivated fetal bovine serum and antibiotics (100 U/ml of penicillin A and 100  U/ml of streptomycin) at 37 ℃ in a humidified incubator containing 5% CO_2_ for 2–4 h to separate fibroblasts and cardiomyocytes, and then treated with adenovirus-TOR2A shRNA (GeneChem Co., Shanghai, China), Ang II (10^–6^ M; Sigma) [28, 30] or losartan (10^–6^ M; Selleck, Shanghai, China) for 24 h.

### MTT assay for cell viability

Seeded cardiomyocytes or fibroblasts in a 96-well plate. According to the ability of living cells which utilized thiazole blue and converted it into purple methazan, the viability of cardiomyocytes was measured by the function of living cells which reduced MTT to methazan. Methazan absorbed 570 nm of light and could be measured by microplate reader (VT, BioTek, USA). The results were normalized to the Saline + Control shRNA group.

### Real-time quantitative polymerase chain reaction (RT-qPCR)

Total RNA was isolated from cardiac tissues or cultured primary cells with Trizol reagents (Invitrogen, USA). The concentration of mRNA was acquired by NANODROP ONE (Thermo, Shanghai, China), and then 0.5 μg RNA was reversely transcribed into cDNA with 10 μl random primers and PrimeScript^™^ RT Master mix (Takara Biotechnology Co., Ltd.). RT-qPCR analysis was performed with ABI prism 7900 sequence detection system (Applied Biosystems, California, USA). All samples were magnified three times in a 384-well plate, and then cycled 40 times for 10 s at 95 ℃ and for 1 min at 60 ℃. The mean cycle threshold (CT) values were normalized to endogenous control (GAPDH) values, and the relative mRNA transcription level was calculated as 2^−ΔΔCt^ in a previous study [31]. The primers were shown in Additional file 1: Table S1.

### Statistical analysis

Data were expressed as the mean ± SEM. GraphPad Prism 7.0 (GraphPad software Inc., CA, USA) was used to analyze data. The differences of parameters between two groups were compared by *t* test, and those among multiple groups were compared by one-way analysis of variance (ANOVA) with the Bonferroni post hoc test. Statistical significance was shown when the two-tailed ^*^P < 0.05, ^**^P < 0.01, ^***^P < 0.001, and ^****^P < 0.0001.

## Results

### Levels of salusins

**Fig. 1:**
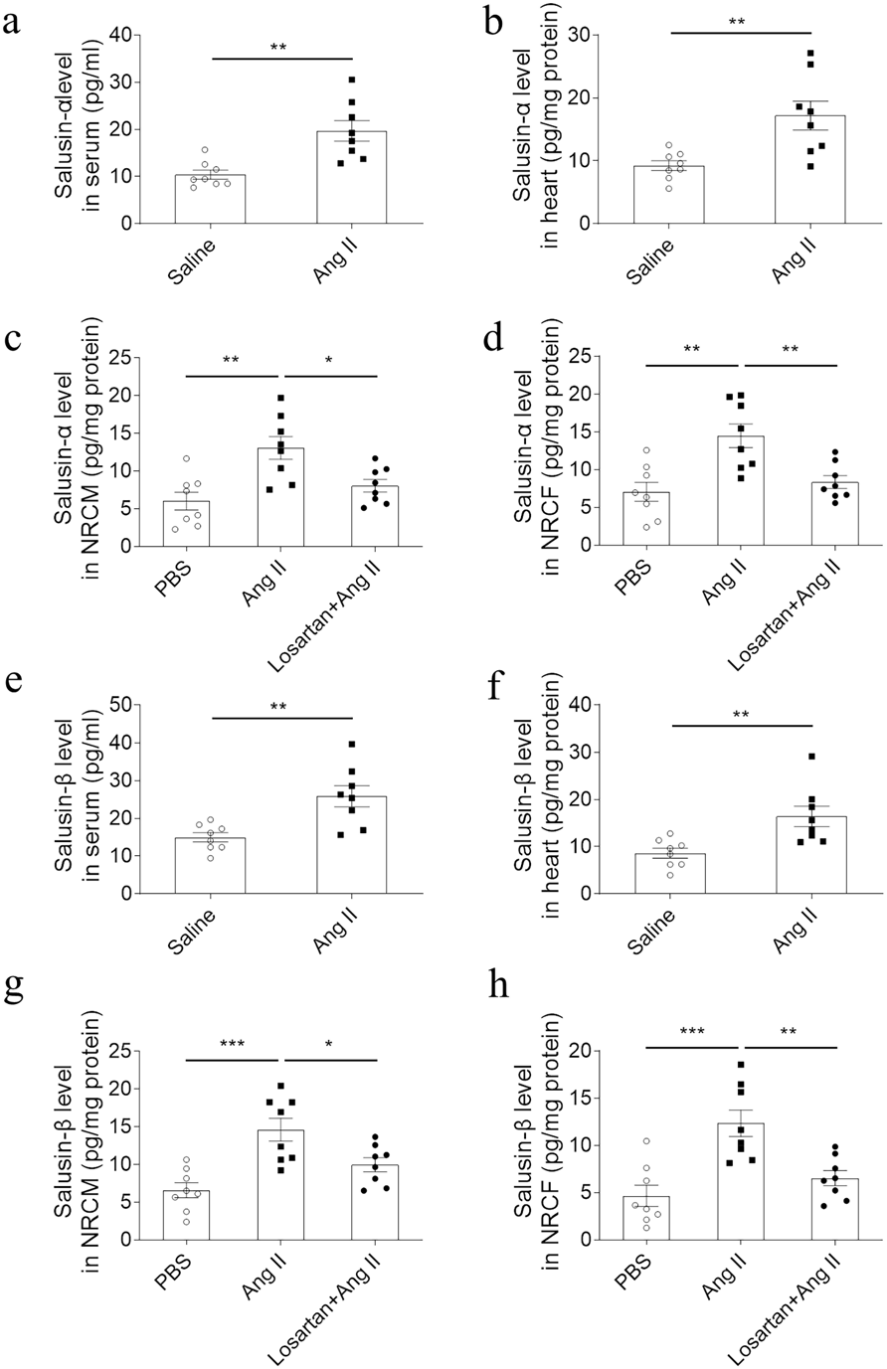
Levels of salusins. Salusin-α levels increased in the serum (**a**) and heart (**b**) of Ang II-infused rats, as well as in the Ang II-treated NRCMs (**c**) and NRCFs (**d**). Salusin-β levels raised in the serum (**e**) and heart (**f**) of Ang II-infused rats, as well as in the Ang II-treated NRCMs (**g**) and NRCFs (**h**). Data are expressed as the mean ± SEM. N = 8 in each group. Ang, angiotensin; NRCMs, neonatal rat cardiomyocytes; NRCFs, neonatal rat cardiac fibroblasts

Salusin-α levels were higher in serum and cardiac tissues of Ang II-infused rats compared with saline controls (Fig. 1a, b). After treatment with Ang II for 24 h, the levels of salusin-α increased in both NRCMs (Fig. 1c) and NRCFs (Fig. 1d), which were reversed after losartan treatment. In addition, similar results of salusin-β were detected in serum and cardiac tissues of Ang II-infused rats (Fig. 1e, f) as well as in both NRCMs (Fig. 1g) and NRCFs (Fig. 1h) after treatment with Ang II, which were reversed after losartan treatment.

### Alleviation of cardiac hypertrophy by TOR2A downregulation

**Fig. 2:**
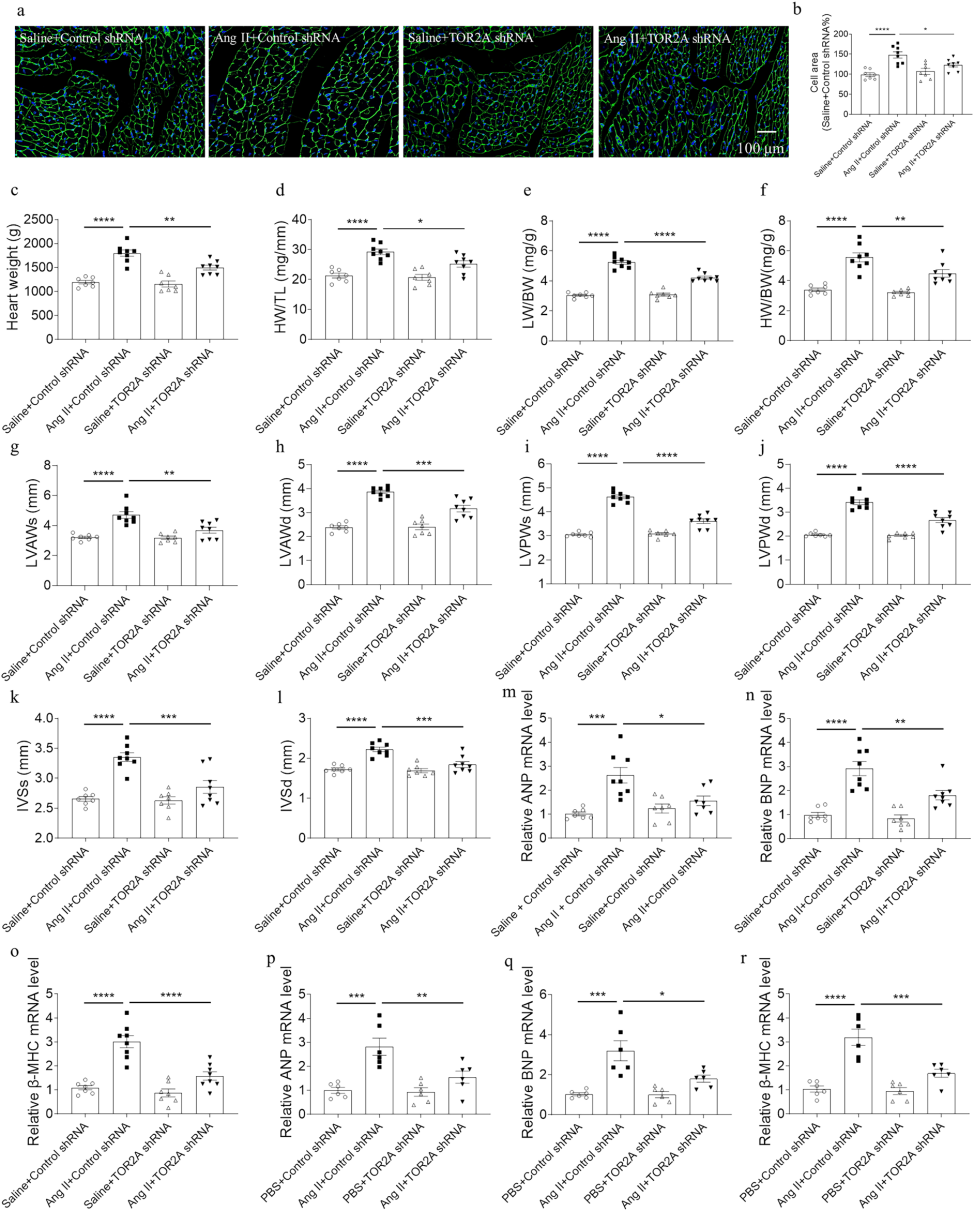
Attenuation of cardiac hypertrophy via TOR2A downregulation. **a**, **b** The increased cardiomyocyte area induced by Ang II was suppressed after TOR2A downregulation. **c**–**l**) The increases of heart weight, HW/BW, HW/TL, LW/BW, LVAWs, LVAWd, LVPWs, LVPWd, IVSs and IVSd in rats infused with Ang II were suppressed after TOR2A downregulation. The increased levels of ANP, BNP and β-MHC in the heart of Ang II-infused rats (**m**–**o**) and Ang II-treated NRCMs (**p**–**r**) were suppressed by TOR2A downregulation. Data are expressed as the mean ± SEM. **a**–**o** Saline +Control shRNA and Saline + TOR2A shRNA groups (n = 7), and Ang II + Control shRNA and Ang II + TOR2A shRNA groups (n = 8). **p**–**r** N = 6 in each group. Scale bar: 100 μm. *Ang* angiotensin, *TOR2A* torsin family 2 member A, *HW* heart weight, *BW* body weight, *TL* tibia length, *LW* left ventricle weight, *LVAWs* left ventricle anterior wall thickness at end systole, *LVAWd* left ventricle anterior wall thickness at end diastole, *LVPWs* left ventricle posterior wall thickness at end systole, *LVPWd* left ventricle posterior wall thickness at end diastole, *IVSs* interventricular septal thickness at end-systole, *IVSd* interventricular septal thickness at end-diastole, *ANP* atrial natriuretic peptide, *BNP* brain natriuretic peptide, *NRCMs* neonatal rat cardiomyocytes

The levels of salusin-α and salusin-β were reduced in the heart of rats after TOR2A knockdown (Additional file 1: Figure S1a). Compared with saline controls, the cardiomyocyte area was enlarged in Ang II-induced cardiac hypertrophy, which was alleviated by TOR2A knockdown (Fig. 2a, b); heart weight, HW/BW, HW/TL, LW/BW, LVAWs, LVAWd, LVPWs, LVPWd, IVSs and IVSd were elevated in Ang II-infused rats and all of them were attenuated by TOR2A downregulation (Fig. 2c-l). There were no significance in the body weight (Additional file 1: Figure S2), EF, FS LVVs, LVVd, LVIDs and LVIDd (Additional file 1: Figure S3a–f). The levels of salusin-α and salusin-β were reduced in the NRCMs after TOR2A knockdown (Additional file 1: Figure S1). In comparison with saline controls, relative transcription level of ANP, BNP and β-MHC was elevated in the cardiac tissues of Ang II-infused rats, which can be suppressed by TOR2A downregulation (Fig. 2m–o). A similar result was detected in the NRCMs after incubation with Ang II for 24 h (Fig. 2p–r). There were no significant differences in the NRCMs survival rate treating with Ang II and TOR2A knockdown through the MTT assay (Additional file 1: Figure S4a).

### Attenuation of cardiac fibrosis by TOR2A downregulation

**Fig. 3:**
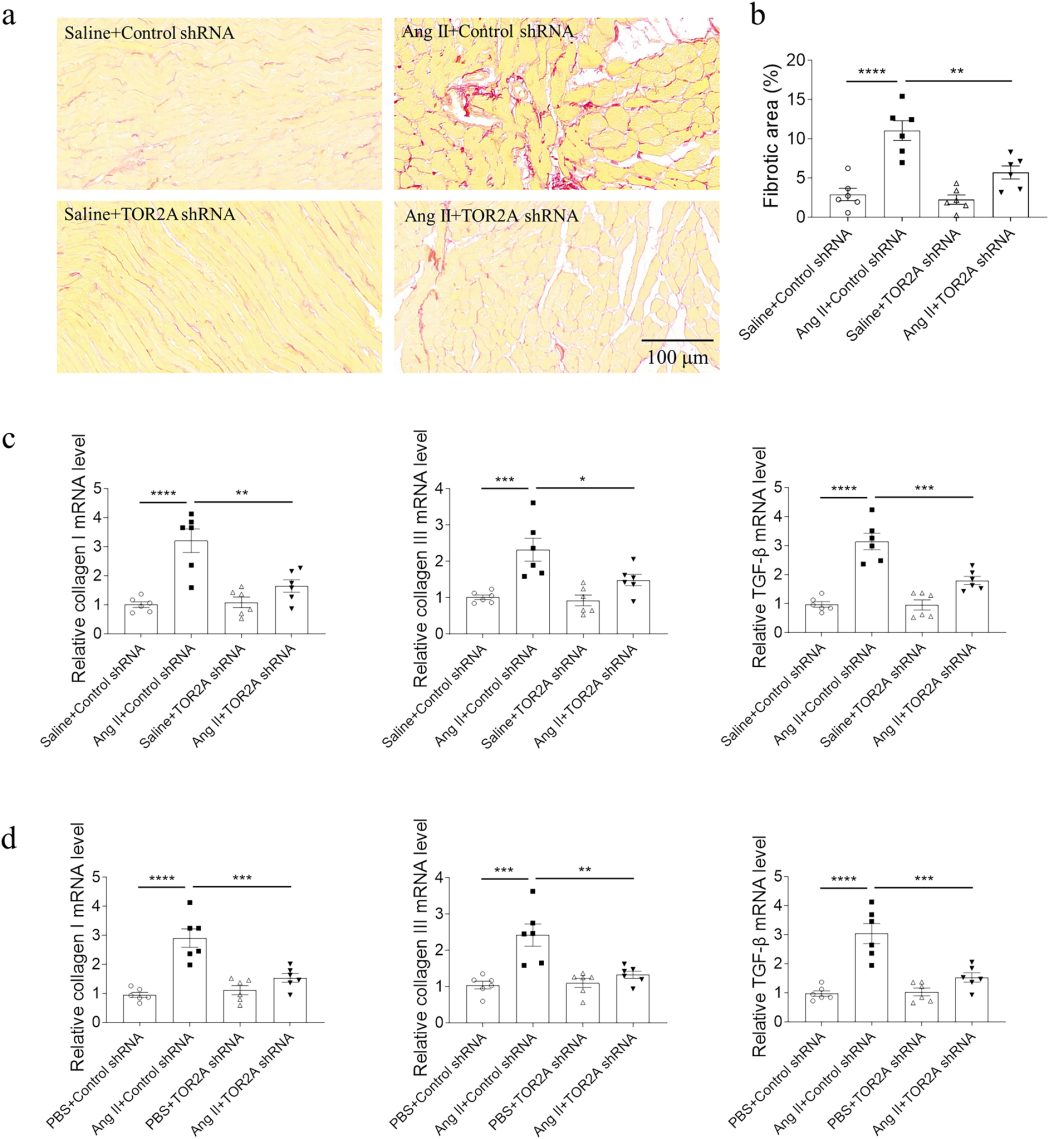
Attenuation of cardiac fibrosis via TOR2A downregulation. **a**–**b** The increase of cardiac fibrosis in the rats infused with Ang II was suppressed by TOR2A downregulation. The increased levels of collagen I, collagen III and TGF-β in the heart of Ang II-infused rats (**c**) and Ang II-treated NRCFs (**d**) were inhibited by TOR2A downregulation. Data are expressed as the mean ± SEM. N = 6 in each group. Scale bar: 100 μm. *Ang* angiotensin, *TOR2A* torsin family 2 member A, *TGF-β* transforming growth factor-beta, *NRCFs* neonatal rat cardiac fibroblasts

The levels of salusin-α and salusin-β were reduced in the NRCFs after TOR2A knockdown (Additional file 1: Figure S1c). Compared with saline controls, downregulation of TOR2A significantly attenuated Ang II-induced cardiac fibrosis (Fig. 3a, b). The cardiac fibrosis was determined indirectly by measuring the levels of collagen I, collagen III and TGF-β, and the results of RT-qPCR showed an increase of these parameters in the cardiac tissues, which were suppressed by TOR2A downregulation (Fig. 3c). Similarly, these transcriptions increased in the NRCFs treated with Ang II, which were inhibited by knockdown of TOR2A (Fig. 3d). There were no significant differences in the NRCFs survival rate treating with Ang II and TOR2A knockdown through the MTT assay (Additional file 1: Figure S4b).

### Alleviation of oxidative stress by TOR2A downregulation

**Fig. 4:**
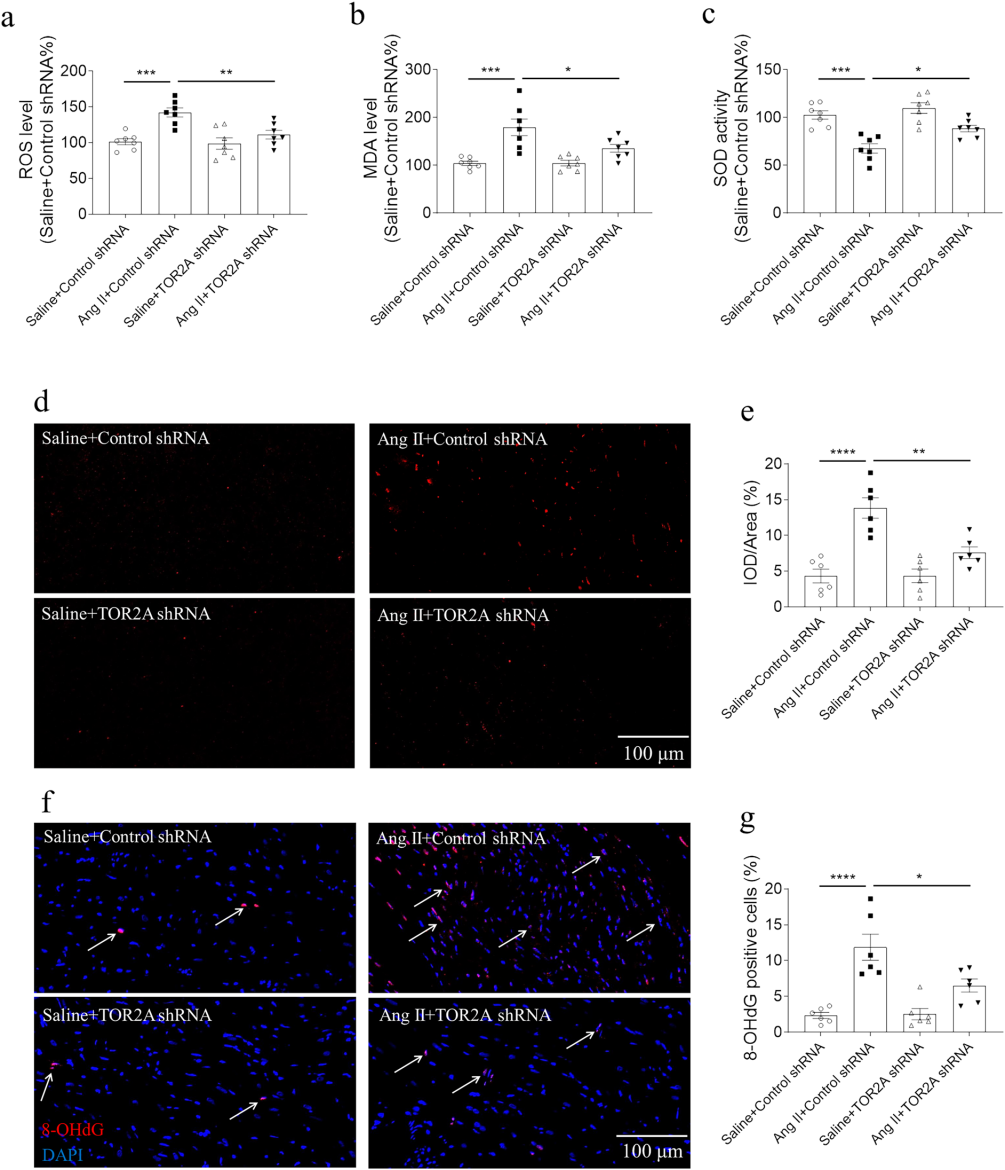
Alleviation of oxidative stress by TOR2A downregulation. The increases of ROS (**a**) and MDA (**b**) in the heart of rats infused with Ang II were inhibited by TOR2A downregulation. **c** The decreased SOD activity in the heart of rats infused with Ang II was reversed after TOR2A downregulation. **d**, **e** The increase of ROS production in the heart of rats infused with Ang II was suppressed by TOR2A downregulation. **f**, **g** The increase of 8-OHdG positive cells in the heart of rats infused with Ang II was blocked after TOR2A downregulation. Data are expressed as the mean ± SEM. **a**–**c** N = 7 in each group. **d**, **g** N = 6 in each group. Scale bar: 100 μm. *Ang* angiotensin, *TOR2A* torsin family 2 member A, *ROS* reactive oxygen species, *MDA* malondialdehyde, *SOD* superoxide dismutase, *8-OHdG* 8-hydroxy-2ʹ-deoxyguanosine

Compared with saline controls, ROS and MDA levels increased in the cardiac tissues of Ang II-infused rats, which was further suppressed by TOR2A knockdown (Fig. 4a, b). Whereas, the activity of SOD decreased in the cardiac tissues of Ang II-infused rats, which was reversed by TOR2A knockdown (Fig. 4c). Fluorescence microscope with single red immunofluorescence showed that, compared with saline control, the capability of ROS production was enhanced in the cardiac tissues of Ang II-infused rats, which was reversed by reducing TOR2A (Fig. 4d, e). In addition, fluorescence microscope with double red/green immunofluorescence demonstrated that, compared with saline control, the 8-OHdG positive cells increased in the cardiac tissues of Ang II-infused rats and it was attenuated by TOR2A knockdown (Fig. 4f, g).

### Alleviation of autophagy by TOR2A downregulation

**Fig. 5:**
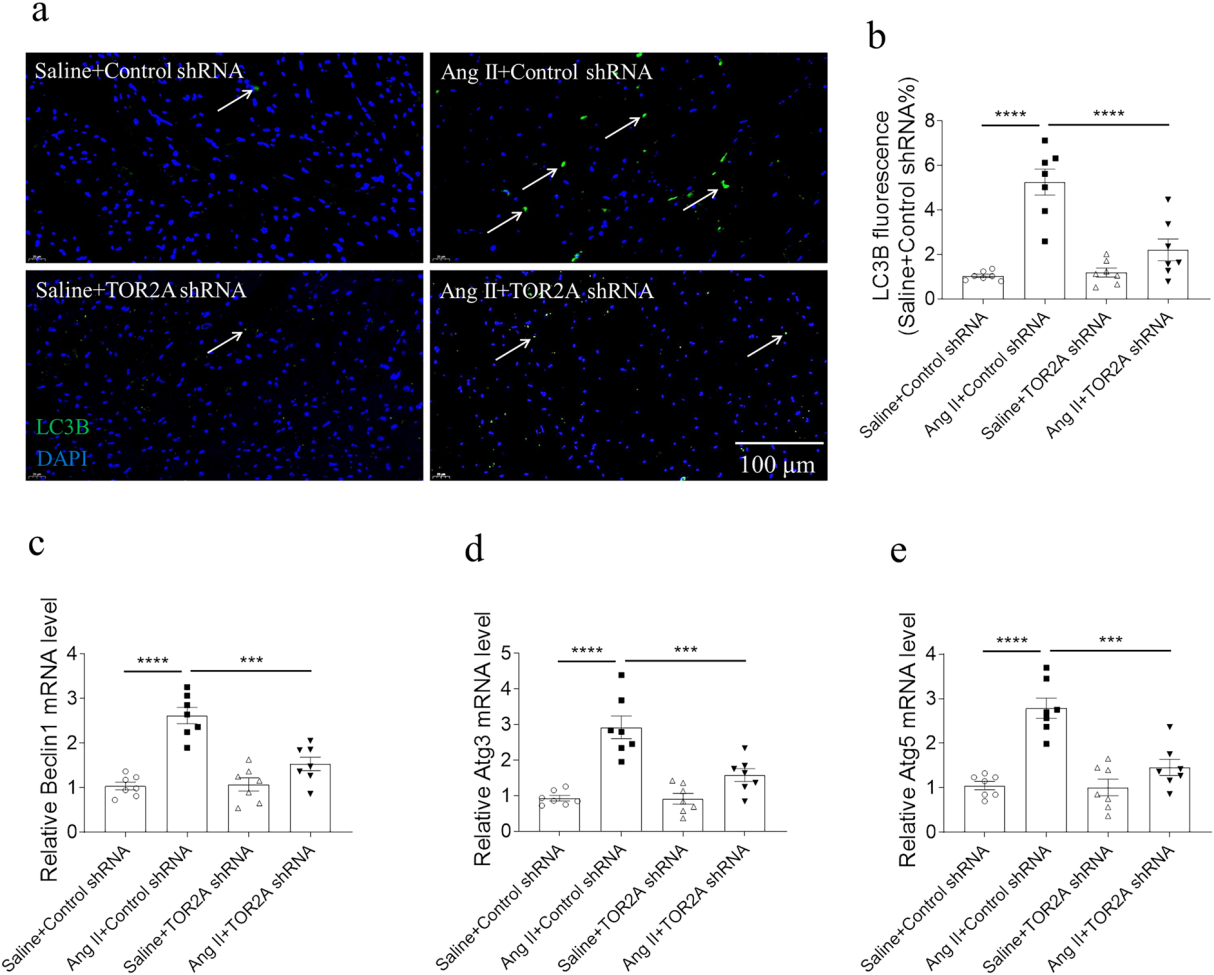
Alleviation of autophagy by TOR2A downregulation. The increases of LC3B (**a**, **b**), Beclin1, Atg3 and Atg5 (**c**-**e**) in the heart of Ang II-infused rats were suppressed by TOR2A downregulation. Data are expressed as the mean ± SEM. N = 7 in each group. Scale bar: 100 μm. *Ang* angiotensin, *TOR2A* torsin family 2 member A, *Atg* autophagy related gene

Fluorescence microscope with double blue/green immunofluorescence suggested that, compared with saline control, LC3B level was higher in the heart of Ang II-infused rats, which was suppressed by TOR2A downregulation (Fig. 5a, b). Simultaneously, the autophagy was determined indirectly by measuring the levels of autophagy-related genes and the results of RT-qPCR revealed that, compared with saline control, the Beclin1 (Fig. 5c), autophagy-related Atg3 (Fig. 5d) and Atg5 increased in the cardiac tissues of Ang II-infused rats, which were suppressed by knockdown of TOR2A.

## Discussion

The primary findings were that the levels of salusin-α and salusin-β increased in rat model with HCM induced by intravenous infusion of Ang II. Notably, downregulation of salusins significantly alleviated Ang II-induced cardiac hypertrophy and fibrosis and targeting salusins attenuated oxidative stress and autophagy. All these findings revealed that downregulation of salusins might alleviate HCM probably via attenuating oxidative stress and autophagy.

Accumulating evidence has documented that salusin-β is involved in the pathogenesis of diabetic cardiomyopathy [21], and knockdown of salusin-β ameliorates hypertension- induced heart failure [32] or myocardial infarction [12]. In the current study, we found that salusin-α and salusin-β levels increased in serum and cardiac tissues of Ang II-infused rats, and knockdown of salusins improved the cardiac hypertrophy in both the heart of Ang II-infused rats and Ang II-treated NRCMs, indicating that knockdown of salusins is conductive to attenuating HCM. Interestingly, we also found that the cardiac fibrosis in the Ang II-infused rats was attenuated by downregulation of salusins, which was supported by the conclusion that salusin-β could promote vascular fibrosis [33]. In addition, treatment with losartan reversed the increases of salusins induced by Ang II, which demonstrated that Ang II is indeed involved in the induction of salusins.

Oxidative stress, a natural imbalance between the ROS production and elimination, plays a certain role in the CVDs [20]. Increased oxidative stress and ROS generation can motivate a variety of transcription factors and protein kinase signaling pathways that are involved in cardiac hypertrophy [34]. Numerous data have demonstrated that oxidative stress was involved regarding the effects of salusins on multiple diseases, including diabetic cardiomyopathy [21], acute ischemic renal failure [35], and atherosclerosis [36]. The present study showed that the increase of oxidative stress in the heart of cardiac hypertrophic rat infused with Ang II was attenuated by downregulation of salusins, suggesting that downregulation of salusins might alleviate HCM via attenuation of oxidative stress.

A potential relationship between autophagy dysregulation and the development and progression of several pathologies, including CVDs, has been found in previous articles [37]. Autophagy activation highly relies on the protein transcription and post-translational regulation of several Atg genes [38, 39]. In the present study, we found that increased Atg3 and Atg5 levels in the heart of rats with Ang II-induced cardiac hypertrophy were suppressed after downregulation of salusins. Meanwhile, knockdown of salusins also inhibited the increase of LC3B and Beclin1 levels in the heart of rats with cardiac hypertrophy. These results unveiled that downregulation of salusins could attenuate HCM possibly through regulation of autophagy.

There are two limitations in the present study. Firstly, only one of the HCM models was used to detect the role of salusin downregulation in hypertrophic cardiomyopathy. Other HCM models will be used to ascertain the protective effect of salusin downregulation on cardiac hypertrophy. Secondly, the mechanism of salusin knockdown on the ROS reduction is not explored in the present study. Previous study showed that salusin increased the level of NOX2 and NOX4 [21], which resulted in ROS increase, and salusin-β IgG reduced NADPH oxidase activity and ROS level [40]. The mechanism of salusin affecting ROS to regulate HCM is an interesting issue and will be explored in our future study.

Collectively, our findings revealed the increased levels of salusins in HCM. Downregulation of salusins improve cardiac hypertrophy and fibrosis, and attenuate the enhanced oxidative stress and autophagy in HCM. Hence, targeting salusins may be a promising treatment strategy for HCM in the future.

### Supplementary Information

**Additional file 1: Figure S1.** Levels of salusins after TOR2A downregulation. **a** Levels of salusin-α and salusin-β were reduced in the heart of rats after TOR2A knockdown. **b** Levels of salusin-α and salusin-β were reduced in the NRCMs after TOR2A knockdown. **c** Levels of salusin-α and salusin-β were reduced in the NRCFs after TOR2A knockdown. **a** Saline + Control shRNA and Saline + TOR2A shRNA groups (n=7), and Ang II + Control shRNA and Ang II + TOR2A shRNA groups (n = 8). **b**, **c** N = 6 in each group. *Ang* angiotensin, *TOR2A* torsin family 2 member A, *NRCMs* neonatal rat cardiomyocytes, *NRCFs* neonatal rat cardiac fibroblasts. **Figure S2.** Levels of body weight. There was no significant difference in the body weight among four groups. Saline + Control shRNA and Saline + TOR2A shRNA groups (n = 7), and Ang II + Control shRNA and Ang II + TOR2A shRNA groups (n = 8). **Figure S3.** Effects of TOR2A downregulation on cardiac function. There was no significant difference in the EF, FS LVVs, LVVd, LVIDs and LVIDd among four groups. Saline + Control shRNA and Saline + TOR2A shRNA groups (n=7), and Ang II + Control shRNA and Ang II + TOR2A shRNA groups (n=8). **Figure S4.** Levels of cell viability. **a** There were no significant differences in the NRCMs survival rate treating with Ang II and TOR2A knockdown. **b** There were no significant differences in the NRCFs survival rate treating with Ang II and TOR2A knockdown. N=6 in each group. *Ang* angiotensin, *TOR2A* torsin family 2 member A, *NRCMs* neonatal rat cardiomyocytes, *NRCFs* neonatal rat cardiac fibroblasts. **Table S1.** List of utilized primers for quantitative real time-PCR (qRT-PCR).

## Data Availability

Available upon requests.
