# Supplementary figures and images for: Downregulation of salusins alleviates hypertrophic cardiomyopathy via attenuating oxidative stress and autophagy
Source: Eur J Med Res. 2024 Feb 9;29:109. doi: 10.1186/s40001-024-01676-z (PMC10854150; doi:10.1186/s40001-024-01676-z)

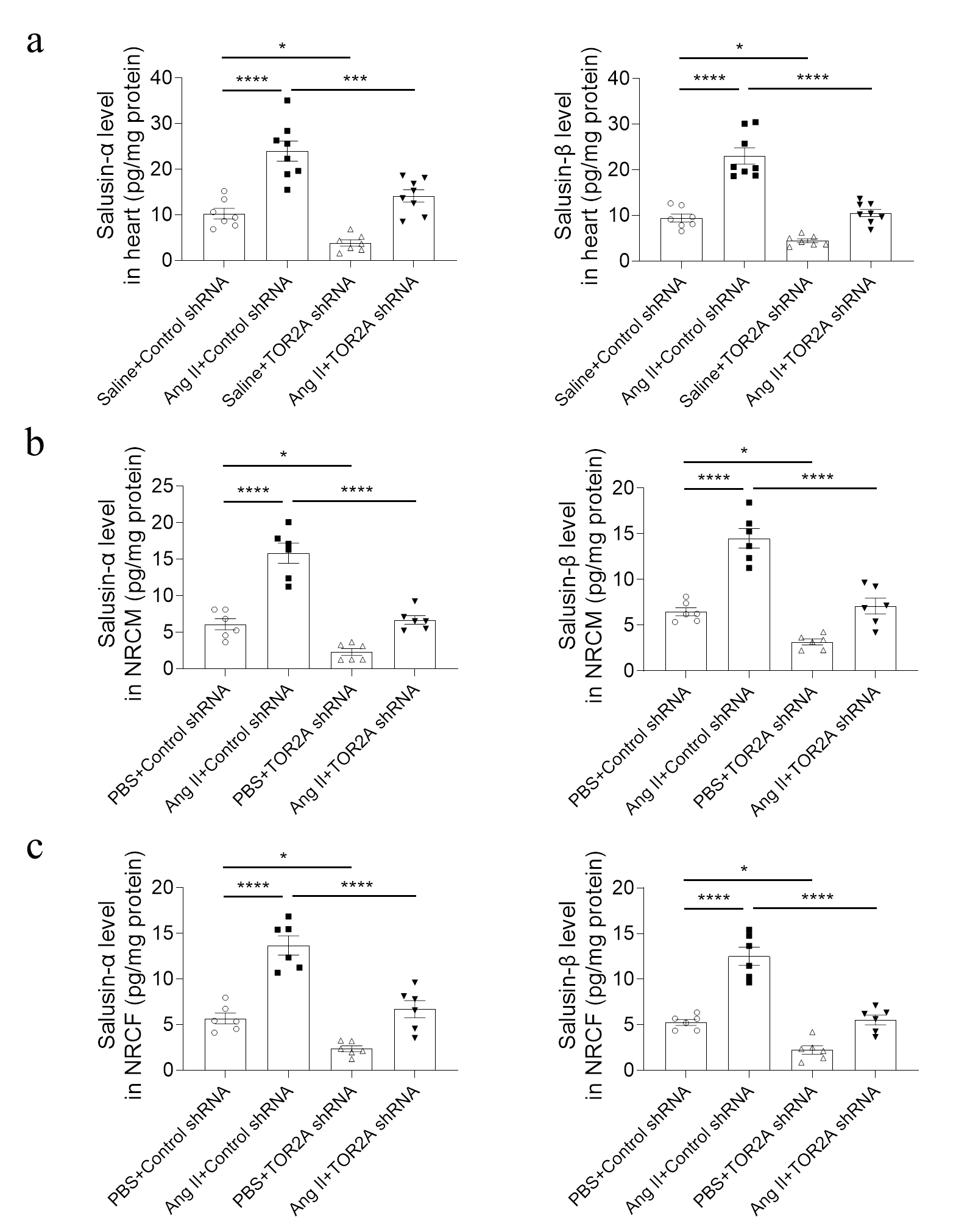

Supplement: Supplementary file 1 — Additional file 1: Figure S1. Levels of salusins after TOR2A downregulation. a Levels of salusin-α and salusin-β were reduced in the heart of rats after TOR2A knockdown. b Levels of salusin-α and salusin-β were reduced in the NRCMs after TOR2A knockdown. c Levels of salusin-α and salusin-β were reduced in the NRCFs after TOR2A knockdown. a Saline + Control shRNA and Saline + TOR2A shRNA groups (n=7), and Ang II + Control shRNA and Ang II + TOR2A shRNA groups (n = 8). b, c N = 6 in each group. Ang angiotensin, TOR2A torsin family 2 member A, NRCMs neonatal rat cardiomyocytes, NRCFs neonatal rat cardiac fibroblasts. Figure S2. Levels of body weight. There was no significant difference in the body weight among four groups. Saline + Control shRNA and Saline + TOR2A shRNA groups (n = 7), and Ang II + Control shRNA and Ang II + TOR2A shRNA groups (n = 8). Figure S3. Effects of TOR2A downregulation on cardiac function. There was no significant difference in the EF, FS LVVs, LVVd, LVIDs and LVIDd among four groups. Saline + Control shRNA and Saline + TOR2A shRNA groups (n=7), and Ang II + Control shRNA and Ang II + TOR2A shRNA groups (n=8). Figure S4. Levels of cell viability. a There were no significant differences in the NRCMs survival rate treating with Ang II and TOR2A knockdown. b There were no significant differences in the NRCFs survival rate treating with Ang II and TOR2A knockdown. N=6 in each group. Ang angiotensin, TOR2A torsin family 2 member A, NRCMs neonatal rat cardiomyocytes, NRCFs neonatal rat cardiac fibroblasts. Table S1. List of utilized primers for quantitative real time-PCR (qRT-PCR). [file 40001_2024_1676_MOESM1_ESM.zip › Figure S1/Figure S1.tif]

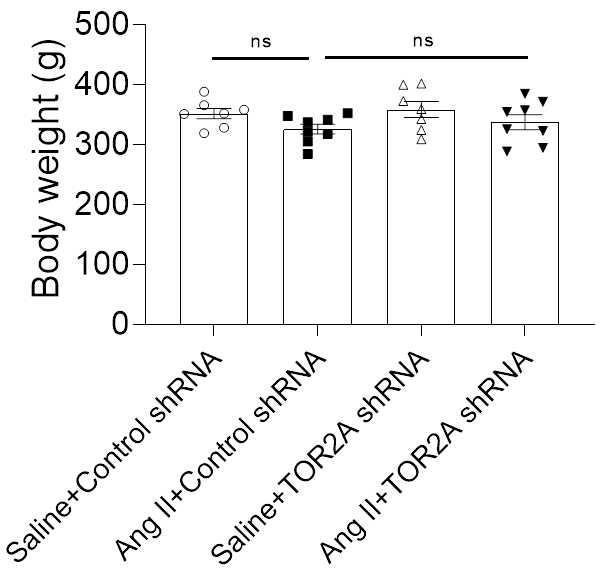

Supplement: Supplementary file 1 — Additional file 1: Figure S1. Levels of salusins after TOR2A downregulation. a Levels of salusin-α and salusin-β were reduced in the heart of rats after TOR2A knockdown. b Levels of salusin-α and salusin-β were reduced in the NRCMs after TOR2A knockdown. c Levels of salusin-α and salusin-β were reduced in the NRCFs after TOR2A knockdown. a Saline + Control shRNA and Saline + TOR2A shRNA groups (n=7), and Ang II + Control shRNA and Ang II + TOR2A shRNA groups (n = 8). b, c N = 6 in each group. Ang angiotensin, TOR2A torsin family 2 member A, NRCMs neonatal rat cardiomyocytes, NRCFs neonatal rat cardiac fibroblasts. Figure S2. Levels of body weight. There was no significant difference in the body weight among four groups. Saline + Control shRNA and Saline + TOR2A shRNA groups (n = 7), and Ang II + Control shRNA and Ang II + TOR2A shRNA groups (n = 8). Figure S3. Effects of TOR2A downregulation on cardiac function. There was no significant difference in the EF, FS LVVs, LVVd, LVIDs and LVIDd among four groups. Saline + Control shRNA and Saline + TOR2A shRNA groups (n=7), and Ang II + Control shRNA and Ang II + TOR2A shRNA groups (n=8). Figure S4. Levels of cell viability. a There were no significant differences in the NRCMs survival rate treating with Ang II and TOR2A knockdown. b There were no significant differences in the NRCFs survival rate treating with Ang II and TOR2A knockdown. N=6 in each group. Ang angiotensin, TOR2A torsin family 2 member A, NRCMs neonatal rat cardiomyocytes, NRCFs neonatal rat cardiac fibroblasts. Table S1. List of utilized primers for quantitative real time-PCR (qRT-PCR). [file 40001_2024_1676_MOESM1_ESM.zip › Figure S1/Figure S2.tif]

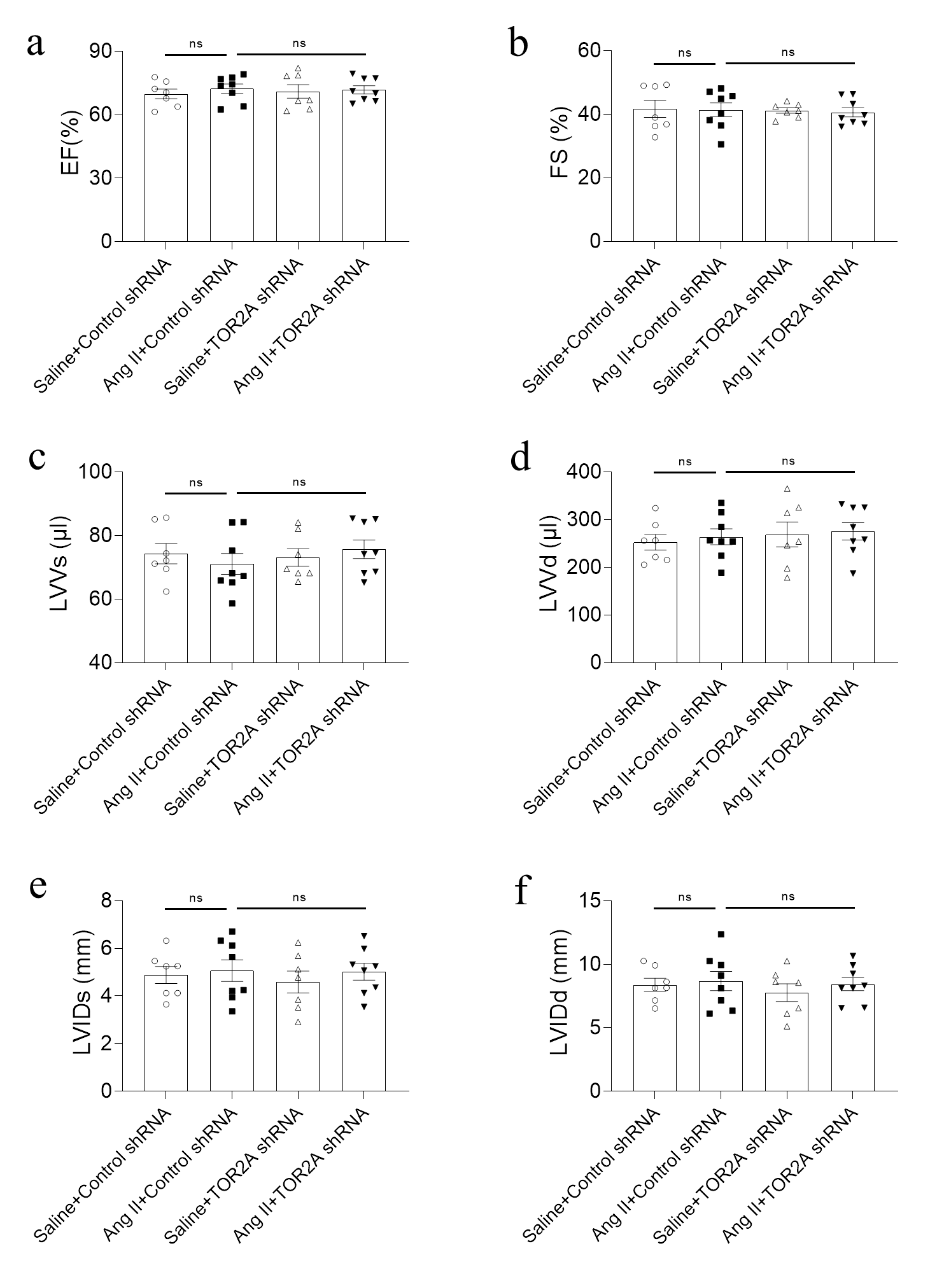

Supplement: Supplementary file 1 — Additional file 1: Figure S1. Levels of salusins after TOR2A downregulation. a Levels of salusin-α and salusin-β were reduced in the heart of rats after TOR2A knockdown. b Levels of salusin-α and salusin-β were reduced in the NRCMs after TOR2A knockdown. c Levels of salusin-α and salusin-β were reduced in the NRCFs after TOR2A knockdown. a Saline + Control shRNA and Saline + TOR2A shRNA groups (n=7), and Ang II + Control shRNA and Ang II + TOR2A shRNA groups (n = 8). b, c N = 6 in each group. Ang angiotensin, TOR2A torsin family 2 member A, NRCMs neonatal rat cardiomyocytes, NRCFs neonatal rat cardiac fibroblasts. Figure S2. Levels of body weight. There was no significant difference in the body weight among four groups. Saline + Control shRNA and Saline + TOR2A shRNA groups (n = 7), and Ang II + Control shRNA and Ang II + TOR2A shRNA groups (n = 8). Figure S3. Effects of TOR2A downregulation on cardiac function. There was no significant difference in the EF, FS LVVs, LVVd, LVIDs and LVIDd among four groups. Saline + Control shRNA and Saline + TOR2A shRNA groups (n=7), and Ang II + Control shRNA and Ang II + TOR2A shRNA groups (n=8). Figure S4. Levels of cell viability. a There were no significant differences in the NRCMs survival rate treating with Ang II and TOR2A knockdown. b There were no significant differences in the NRCFs survival rate treating with Ang II and TOR2A knockdown. N=6 in each group. Ang angiotensin, TOR2A torsin family 2 member A, NRCMs neonatal rat cardiomyocytes, NRCFs neonatal rat cardiac fibroblasts. Table S1. List of utilized primers for quantitative real time-PCR (qRT-PCR). [file 40001_2024_1676_MOESM1_ESM.zip › Figure S1/Figure S3.tif]

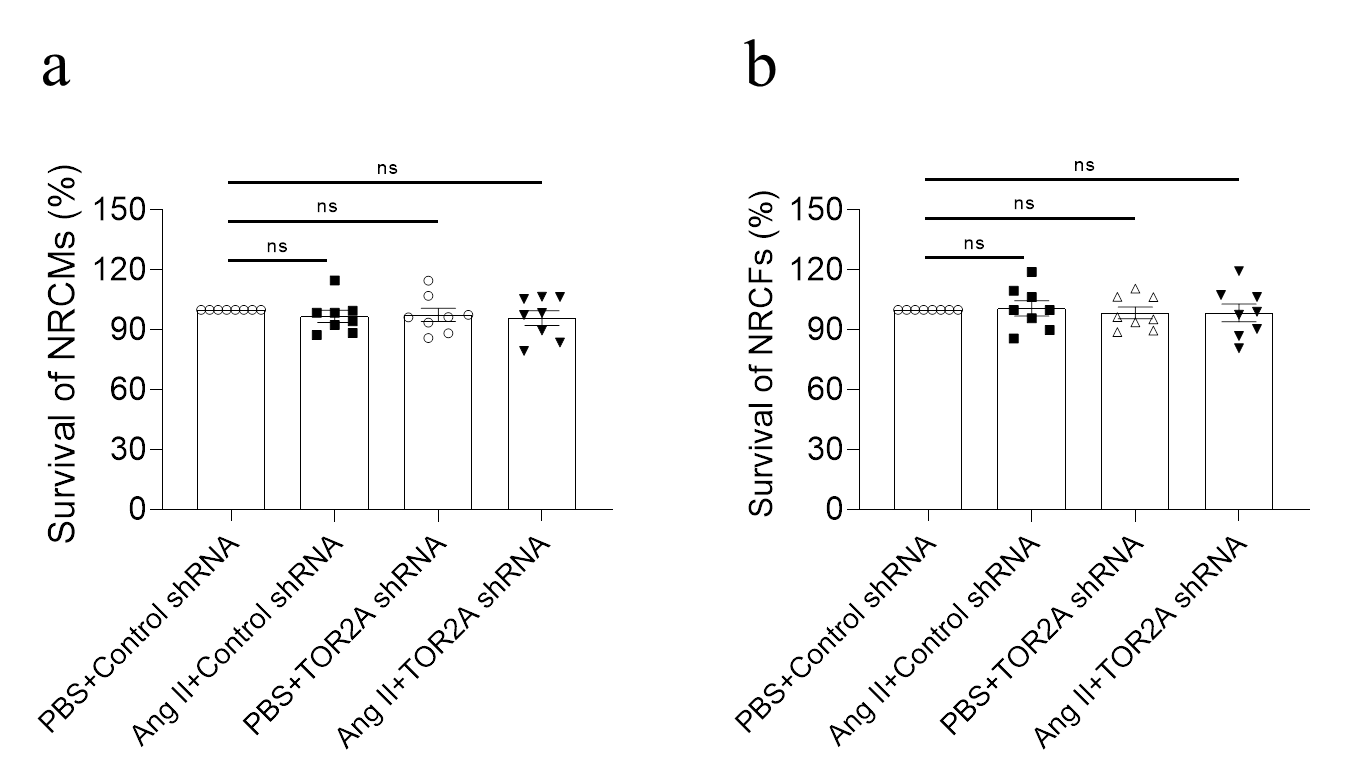

Supplement: Supplementary file 1 — Additional file 1: Figure S1. Levels of salusins after TOR2A downregulation. a Levels of salusin-α and salusin-β were reduced in the heart of rats after TOR2A knockdown. b Levels of salusin-α and salusin-β were reduced in the NRCMs after TOR2A knockdown. c Levels of salusin-α and salusin-β were reduced in the NRCFs after TOR2A knockdown. a Saline + Control shRNA and Saline + TOR2A shRNA groups (n=7), and Ang II + Control shRNA and Ang II + TOR2A shRNA groups (n = 8). b, c N = 6 in each group. Ang angiotensin, TOR2A torsin family 2 member A, NRCMs neonatal rat cardiomyocytes, NRCFs neonatal rat cardiac fibroblasts. Figure S2. Levels of body weight. There was no significant difference in the body weight among four groups. Saline + Control shRNA and Saline + TOR2A shRNA groups (n = 7), and Ang II + Control shRNA and Ang II + TOR2A shRNA groups (n = 8). Figure S3. Effects of TOR2A downregulation on cardiac function. There was no significant difference in the EF, FS LVVs, LVVd, LVIDs and LVIDd among four groups. Saline + Control shRNA and Saline + TOR2A shRNA groups (n=7), and Ang II + Control shRNA and Ang II + TOR2A shRNA groups (n=8). Figure S4. Levels of cell viability. a There were no significant differences in the NRCMs survival rate treating with Ang II and TOR2A knockdown. b There were no significant differences in the NRCFs survival rate treating with Ang II and TOR2A knockdown. N=6 in each group. Ang angiotensin, TOR2A torsin family 2 member A, NRCMs neonatal rat cardiomyocytes, NRCFs neonatal rat cardiac fibroblasts. Table S1. List of utilized primers for quantitative real time-PCR (qRT-PCR). [file 40001_2024_1676_MOESM1_ESM.zip › Figure S1/Figure S4.tif]
